# Curcumin Combined with Flaxseed Oil Modulates Lipid Metabolism in Hamsters Fed a High-Fat, High-Cholesterol Diet: Insights from Lipidomics

**DOI:** 10.3390/nu18111747

**Published:** 2026-05-29

**Authors:** Pin-Hui Wei, Chi-Chang Huang, Yi-Ju Hsu, Yi-Tung Lin, Pei Yu Loe, Wan-Chun Chiu, Shih-Yi Huang

**Affiliations:** 1 School of Nutrition and Health Sciences, College of Nutrition, Taipei Medical University, Taipei 11031, Taiwan; 86022@w.tmu.edu.tw (P.-H.W.); ma07114001@tmu.edu.tw (Y.-T.L.); da07113001@tmu.edu.tw (P.Y.L.); 2 Department of Nutrition, Wan Fang Hospital, Taipei Medical University, Taipei 116035, Taiwan; 3 Graduate Institute of Sports Science, National Taiwan Sport University, Taoyuan 333325, Taiwan; john5523@ntsu.edu.tw (C.-C.H.); ruby780202@ntsu.edu.tw (Y.-J.H.); 4 Research Center of Geriatric Nutrition, College of Nutrition, Taipei Medical University, Taipei 11031, Taiwan; 5 Graduate Institute of Metabolism and Obesity Sciences, Taipei Medical University, Taipei 11031, Taiwan; 6 Nutrition Research Center, Taipei Medical University Hospital, Taipei 11031, Taiwan

**Keywords:** curcumin, flaxseed oil, high-fat, high-cholesterol, lipid metabolism, lipidomics

## Abstract

**Background/Objectives:** Dyslipidemia and hepatic lipid buildup are key features of cardiometabolic disorders caused by high-fat, high-cholesterol diets. Both curcumin and flaxseed oil have been shown to improve lipid metabolism through different mechanisms. This study examined the effects of combining curcumin with flaxseed oil on lipid metabolism in hamsters fed a high-fat, high-cholesterol diet and further investigated the underlying mechanisms using liver and serum lipidomic analyses. **Methods:** Thirty-two male Golden Syrian hamsters were randomly divided into four groups (*n* = 8 per group): control, high-fat/high-cholesterol diet (HFD), HFD with low-dose curcumin–flaxseed oil mixture, and HFD with high-dose curcumin–flaxseed oil mixture. After 8 weeks, serum lipid profiles, hepatic triglyceride (TG) and total cholesterol (TC), fecal TG and TC excretion, hepatic mRNA expression of SREBP-1, ACC, and FAS, and untargeted lipidomic profiles in serum and liver were analyzed. **Results:** Compared with the HFD group, curcumin–flaxseed oil supplementation significantly reduced serum TG, TC, and LDL-C levels, while HDL-C remained unchanged. Hepatic TG and TC accumulation also decreased significantly, accompanied by increased fecal TG and TC excretion, with a more pronounced effect in the high-dose group. Hepatic SREBP-1 and ACC mRNA expression increased in the low-dose group, whereas FAS expression remained unchanged. Lipidomic analysis showed notable remodeling of diacylglycerol species in both liver and serum. A similar trend was observed in serum TG profiles, particularly TG 54:1 and TG 52:2, suggesting that changes in circulating lipids may mirror the hepatic lipidomic response. **Conclusions:** Curcumin combined with flaxseed oil improved dyslipidemia and hepatic lipid accumulation in hamsters fed a high-fat, high-cholesterol diet, potentially through increased lipid excretion and modulation of hepatic and circulating lipid profiles.

## 1. Introduction

Cardiovascular disease (CVD) remains the leading cause of death worldwide and is closely linked to dyslipidemia and ectopic lipid deposition. Hepatic lipid buildup is a key aspect of metabolic dysfunction-associated steatotic liver disease (MASLD), which is now recognized as a common metabolic disorder strongly associated with increased cardiovascular risk [1]. A Western diet is often marked by higher intake of fat and cholesterol, and long-term adherence to this eating pattern may disturb lipid balance, leading to increased circulating triglycerides (TG) and cholesterol levels, and causing lipid buildup in the liver. This lipid overload further boosts hepatic de novo lipogenesis (DNL) and cholesterol production, while reducing lipid use and metabolic stability, which contributes to the development of metabolic problems [2,3]. SREBP-1 is a key regulator of hepatic lipogenesis and activates downstream genes such as acetyl-CoA carboxylase (ACC) and fatty acid synthase (FAS) [4]. Studies report increased hepatic SREBP-1c under high-fat feeding, although opposite findings have been observed in some models, especially for SREBP-2 under chronic cholesterol-rich conditions [5]. Therefore, the regulation of the de novo lipogenesis biosynthetic pathway may be influenced by multiple factors related to dietary composition.

Curcumin, the primary bioactive polyphenol from *Curcuma longa*, has been extensively researched for its beneficial effects on lipid metabolism and inflammation reduction. Studies show that curcumin can enhance dyslipidemia and hepatic steatosis by inhibiting pathways involved in fat creation and cholesterol production, including SREBP-1-mediated lipogenesis and cholesterol metabolism, while also decreasing liver inflammation [6,7]. In contrast, flaxseed oil is rich in α-linolenic acid (ALA), a plant-based *n*-3 polyunsaturated fatty acid, and has been shown to improve lipid balance by reducing triglyceride and cholesterol levels and affecting liver lipid metabolism. Treatment with flaxseed oil alone significantly improved the lipid profile and inflammatory markers and also greatly decreased oxidative stress levels [8]. Given that curcumin is fat-soluble and its absorption may be enhanced when consumed with a fatty matrix, combining curcumin with flaxseed oil may provide a practical dietary strategy for evaluating their complementary effects on lipid metabolic regulation.

Traditional biochemical measurements offer limited insights and cannot fully capture the complex lipid remodeling caused by dietary interventions. Lipidomics, a mass spectrometry-based platform that thoroughly analyzes lipid species in biological systems, has become a valuable tool for assessing changes in lipid metabolism and identifying molecular lipid signatures related to nutritional interventions [9,10]. In particular, the liver lipidome can directly reflect hepatic lipid deposition and metabolic remodeling, whereas the plasma lipidome may reveal systemic lipid disturbances associated with metabolic dysfunction [11].

Syrian golden hamsters are sensitive to dietary fat and cholesterol and are widely used in studies of diet-induced dyslipidemia and hepatic lipid accumulation [12]. Compared with commonly used rodents such as mice or rats, hamsters possess several lipid and lipoprotein metabolic characteristics that are more comparable to humans, including Cholesteryl ester transfer protein (CETP) activity, hepatic apolipoprotein B-100 secretion, intestinal apolipoprotein B-48 secretion, receptor-mediated LDL cholesterol uptake, and responsiveness to diet-induced hyperlipidemia [13]. Under high-fat and high-cholesterol dietary conditions, hamsters can develop hyperlipidemia, hepatic lipid deposition, impaired glucose metabolism, and MASLD-like hepatic alterations. Previous studies have also shown that curcumin improves hyperlipidemia, insulin resistance, and hepatic lipid accumulation in high-fat-fed hamsters [14], while flaxseed oil attenuates serum lipid abnormalities and nonalcoholic fatty liver changes in hyperlipidemic hamster [15]. Therefore, this model was considered appropriate for evaluating the effects of the curcumin and flaxseed oil mixture on diet-induced lipid dysregulation and hepatic lipid accumulation. The current study aimed to examine the effects of curcumin combined with flaxseed oil on lipid metabolism in hamsters fed a high-fat, high-cholesterol diet, focusing specifically on dyslipidemia and hepatic lipid accumulation. By integrating metabolic profiling and hepatic lipidomics with lipid molecular markers of fibrosis and inflammation, we provide mechanistic insights into the interplay between dietary modulation in managing obesity-related hepatopathy. Thus, the study sought to explore the potential mechanisms behind these effects through liver and plasma lipidomic analyses.

## 2. Materials and Methods

### 2.1. Animal Protocol and Experimental Design

Thirty-two male Golden Syrian hamsters (8 weeks old) were randomly divided into four groups of eight. The control group was fed the AIN-93M basal diet (without added cholesterol; fat content 4%, *w*/*w*) and given water daily by gavage as the vehicle. The other three groups received a high-fat diet (HFD) (Table 1) supplemented with cholesterol to induce hyperlipidemia. This diet contained 15% fat (*w*/*w*) and 0.2% cholesterol (*w*/*w*) [16]. Of these three high-fat diet groups, two received daily oral gavage of either a low or high dose of the curcumin–flaxseed oil mixture as the intervention. The experiment lasted for eight weeks. Curcumin is a lipophilic compound with limited aqueous solubility and poor oral bioavailability. Therefore, curcumin was administered together with flaxseed oil as an oil-based dietary matrix, as previous studies have suggested that oil-based curcuminoid formulations may provide better curcuminoid bioavailability than non-oil-based powdered formulations [17]. The dose of the curcumin and flaxseed oil mixture was adjusted weekly according to the mean body weight of each group to maintain a consistent dose throughout the intervention period, and body weight was monitored weekly to minimize the influence of body weight differences on dose administration. The doses were calculated based on the recommended human intake of one capsule per day (380 mg/capsule) for a 60 kg adult, corresponding to 6.33 mg/kg body weight/day of the formulation as the low dose, and three capsules daily were the high dose. Using a human-to-hamster conversion factor of 7.4, the doses for hamsters were 46.84 mg/kg/day for the low dose (L) and 140.53 mg/kg/day for the high dose (H) [18]. All animal procedures were approved by the Institutional Animal Care and Use Committee of Taipei Medical University (approval number: LAC2023-0098).

### 2.2. Sample Collection

Animals were provided ad libitum access to food and water throughout the experimental period. Food intake and water consumption were recorded daily, and body weight was measured weekly. Fecal samples were collected in the last three days before sacrifice, stored at −80 °C, and then freeze-dried and ground before analysis. At the end of the experiment, hamsters were deeply anesthetized with Zoletil plus xylazine (50/10 mg/kg body weight, i.p.). Blood was collected by cardiac puncture, and the animals were subsequently euthanized by exsanguination under deep anesthesia. The blood samples were taken and centrifuged at 1500× *g* for 15 min at room temperature to obtain serum. The liver, serum, and fecal samples were collected and stored at −80 °C for subsequent analysis.

### 2.3. Serum AST/ALT and Lipid Profile Analysis

Serum aspartate aminotransferase (AST) activity was measured using an AST Activity Assay Kit (E-BC-K236-S, Elabscience, Houston, TX, USA), and serum alanine aminotransferase (ALT) activity was measured using an ALT Activity Assay Kit (E-BC-K235-M, Elabscience, Houston, TX, USA), according to the manufacturer’s instructions. AST and ALT activities were expressed as U/L and used as biochemical indicators of hepatic injury. Serum triglycerides (TG), total cholesterol (TC), high-density lipoprotein cholesterol (HDL-C), and low-density lipoprotein cholesterol (LDL-C) were measured using commercially available enzymatic colorimetric assay kits and analyzed with an automated biochemical analyzer (Hitachi 7060, Hitachi, Tokyo, Japan). The kits used were DiaSys Triglycerides FS, DiaSys Cholesterol FS, HDL-C Direct FS, and LDL-C Direct FS kits (DiaSys Diagnostic Systems GmbH, Holzheim, Germany). The analyzer was calibrated according to the manufacturer’s instructions before analysis. Low- and high-level quality control sera were included in each analytical batch to ensure assay reliability. The intra- and inter-assay coefficients of variation were both below 5%, according to the manufacturer’s specifications.

### 2.4. Hepatic and Fecal Triglyceride and Cholesterol Analysis

#### 2.4.1. Hepatic Lipid Extraction and Analysis

Liver tissue (100 mg) was homogenized in 1 mL of mixed solvent (chloroform:isopropanol:NP40 = 7:11:0.1, *v*/*v*/*v*) and centrifuged at 15,000× *g* for 10 min at room temperature. The supernatant (400 μL) was collected and dried in a vacuum concentrator at 50 °C for 30 min. The lipid residue was reconstituted with buffer solution. The hepatic lipid extract was homogenized using an ultrasonic cleaner (Branson 5800, Branson Ultrasonics, Brookfield, CT, USA) and mixed thoroughly with a vortex mixer (Vortex-Genie II, Scientific Industries, Bohemia, NY, USA) before lipid measurement. Hepatic triglyceride (TG) and total cholesterol (TC) levels were measured using commercially available enzymatic colorimetric assay kits, including the GPO-PAP method kit (TR1697, Randox Laboratories Ltd., Crumlin, Antrim, UK) and the CHOD-PAP method kit (CH3810, Randox Laboratories Ltd., Crumlin, Antrim, UK), according to the manufacturers’ instructions.

#### 2.4.2. Fecal Lipid Extraction and Analysis

Dried and ground fecal samples (100 mg) were extracted with 1 mL of chloroform:methanol (2:1, *v*/*v*). The mixture was filtered through filter paper, and the filtrate was collected and dried using a vacuum concentrator. The dried lipid residue was reconstituted in dimethyl sulfoxide (DMSO). The fecal lipid extract was homogenized using an ultrasonic cleaner (Branson 5800, Branson Ultrasonics, Brookfield, CT, USA) and mixed thoroughly with a vortex mixer (Vortex-Genie II, Scientific Industries, Bohemia, NY, USA) before lipid determination. Fecal triglyceride (TG) and total cholesterol (TC) levels were measured using the GPO-PAP method kit (TR1697, Randox Laboratories Ltd., Crumlin, Antrim, UK) and the CHOD-PAP method kit (CH3810, Randox Laboratories Ltd., Crumlin, Antrim, UK), according to the manufacturers’ instructions.

### 2.5. Hepatic Gene Expression Analysis by RT-qPCR

Total RNA was isolated from liver tissues using TRIzol reagent (Thermo Fisher Scientific, Cat. No. 15596018, Waltham, MA, USA) and reverse-transcribed into complementary DNA (cDNA) using the RevertAid First Strand cDNA Synthesis Kit (Thermo Fisher Scientific, Cat. No. K1621, Waltham, MA, USA). Quantitative real-time PCR (RT-qPCR) was carried out using Maxima SYBR Green/ROX qPCR Master Mix (2×) (Thermo Fisher Scientific, Cat. No. K0221, Waltham, MA, USA) on a QuantStudio™ 1 Real-Time PCR System (Applied Biosystems, Waltham, MA, USA).

The relative mRNA expression levels of SREBP-1c, ACC, and FAS were determined using gene-specific primers and normalized to β-actin (ACTB) (Table 2). Relative expression was calculated using the 2^−ΔΔCt^ method and presented as fold changes compared with the control group.

### 2.6. Lipidomics Analysis

#### 2.6.1. Lipid Extraction and Internal Standards

Lipids from serum or liver tissues were extracted following the Folch method [19]. To improve the reliability and reproducibility of the lipidomics procedure, two exogenous lipid standards were added to each sample to assess lipid recovery during sample preparation and to monitor the stability and variability of instrumental signals. The internal standards used were C17 ceramide (d18:1/17:0) (#860517, Avanti Polar Lipids, Alabaster, AL, USA) and glucosylsphingosine (#HY-N7745, MedChemExpress, Monmouth Junction, NJ, USA).

#### 2.6.2. UPLC-Q-TOF/MS Analysis

After extraction, lipid samples were subjected to untargeted lipidomics analysis using an ultra-high-performance liquid chromatography system (ACQUITY UPLC, Waters, Milford, MA, USA) coupled with a hybrid quadrupole orthogonal time-of-flight mass spectrometer (SYNAPT G2 HDMS, Waters, Milford, MA, USA) with a CSH C18 column operated at 65 °C and a flow rate of 400 μL/min.

#### 2.6.3. Data Processing and Lipid Annotation

The raw UPLC-MS/MS data were processed with the support of bioinformatics software and databases to identify lipid species and their potential functions. Progenesis QI software 3.0 (Waters, Milford, MA, USA) was used for data calibration, peak alignment, and feature screening. The mass tolerance for precursor and fragment ions was set at 15 ppm. Lipid molecules were annotated using the Human Metabolome Database (HMDB). Only features with a score > 30, relative abundance > 60%, and no missing values were included in the subsequent statistical analysis.

### 2.7. Statistical Analysis

Statistical analyses were performed using GraphPad Prism 9.0 software (GraphPad Software, San Diego, CA, USA). Data are presented as mean ± standard deviation (SD). Data normality was assessed using the Shapiro–Wilk test, and homogeneity of variance was evaluated using the Brown–Forsythe test before parametric analysis. For normally distributed data with acceptable variance homogeneity, one-way ANOVA followed by Tukey’s post hoc multiple comparison test was performed. A *p*-value < 0.05 was considered statistically significant.

Lipidomics data were processed and analyzed using the MetaboAnalyst 6.0 platform. Lipid species with more than 20% missing values were excluded, and the remaining missing values were imputed using the k-nearest neighbor (KNN) method at the feature-wise level. Group differences were evaluated by one-way ANOVA with false discovery rate (FDR) correction for multiple testing, and an adjusted *p* value (FDR) < 0.05 was considered statistically significant.

Multivariate analysis using partial least squares discriminant analysis (PLS-DA) and univariate fold change analysis were performed to evaluate differences in relative lipid abundance among the experimental groups. PLS-DA was used to visualize global lipidomic patterns and group separation based on hepatic and serum TG and DG profiles. Model performance was assessed by 5-fold cross-validation using R^2^, Q^2^, and classification accuracy, and potential overfitting was evaluated using 1000-time permutation tests (Figures S1–S4). Differential lipid species were further interpreted together with lipid species–level statistical analyses (Table S1).

## 3. Results

### 3.1. Effects of High-Fat High-Cholesterol Diet and Curcumin–Flaxseed Oil Mixture on Food Intake and Body Weight

**Figure 1:**
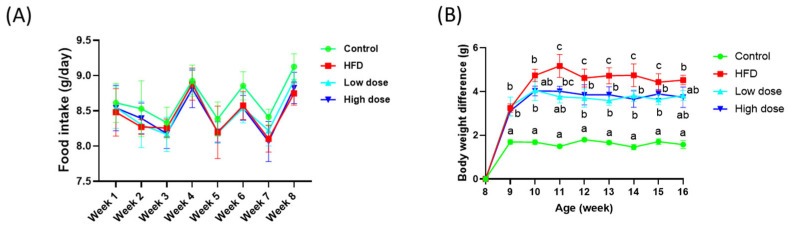
Weekly changes in (**A**) food intake and (**B**) body weight in four experimental groups. Control: normal diet; HFD: high-fat/high-cholesterol diet. Low- and high-dose groups received HCD with supplement (46.84 and 140.53 mg/kg/day, respectively). Data are presented as mean ± SD (*n* = 8 per group). Different letters indicate significant differences among groups (*p* < 0.05).

No statistically significant differences in weekly mean food intake were observed among the groups (Figure 1A). However, mean energy intake was significantly higher in the high-fat, high-cholesterol diet group than in the control group, reflecting the higher caloric density of the HFD compared with the control diet. The control diet provided 3.9 kcal/g, whereas the HFD provided 4.645 kcal/g; therefore, mean energy intake was calculated according to weekly food intake and the energy content of each diet. At baseline (week 8), no significant differences in body weight change were observed among the groups. From week 9 onward, body weight increased in all HFD-fed groups, with the HFD group showing the greatest gain throughout the study period. In contrast, the Control group exhibited the smallest increase in body weight. The low-dose and high-dose intervention groups showed intermediate increases, with values generally lower than those of the HFD group after the intervention began. This pattern became more apparent after week 12, when both intervention groups consistently showed lower body weight changes than the HFD group. Collectively, these findings indicate that the intervention partially attenuated HFD-induced body weight gain, although it did not fully normalize body weight change to the level of the Control group (Figure 1B).

### 3.2. Effects of Curcumin–Flaxseed Oil Mixture on Serum AST and ALT

**Figure 2:**
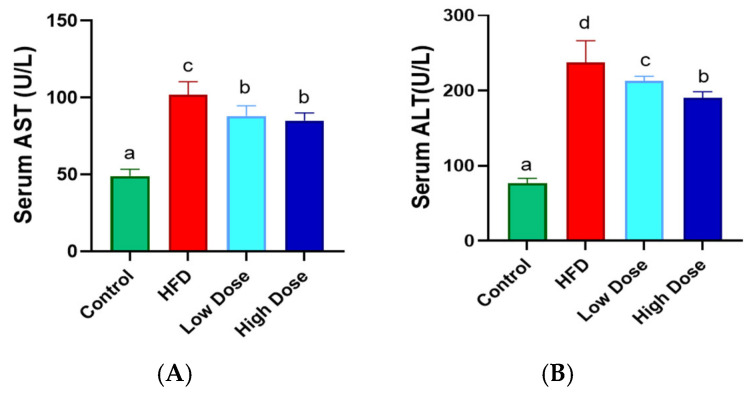
Effects of curcumin–flaxseed oil mixture on the serum AST and ALT of hamsters after the 8-week experimental period. (**A**) Serum AST; (**B**) serum ALT. Control: normal diet; HFD: high-fat/high-cholesterol diet. Low- and high-dose groups received HCD with supplement (46.84 and 140.53 mg/kg/day, respectively). Values are presented as mean ± SD (*n* = 8 per group). Statistical analysis was performed using one-way ANOVA followed by Tukey’s post hoc test. Different letters indicate significant differences among groups (*p* < 0.05).

Serum AST and ALT levels were increased in the HFD group compared with the Control group, suggesting that the high-fat, high-cholesterol diet induced hepatic injury or increased hepatic stress. Supplementation with curcumin combined with flaxseed oil reduced serum AST and ALT levels compared with the HFD group. In particular, the low-dose group showed significantly lower AST and ALT levels than the HFD group, while the high-dose group exhibited a further reduction in ALT compared with the low-dose group (Figure 2A,B). These findings suggest that curcumin combined with flaxseed oil may help attenuate diet-induced hepatic injury, as reflected by improved serum liver enzyme profiles (Figure 2A,B).

### 3.3. Effects of Curcumin–Flaxseed Oil Mixture on Serum Lipid Profiles

**Figure 3:**
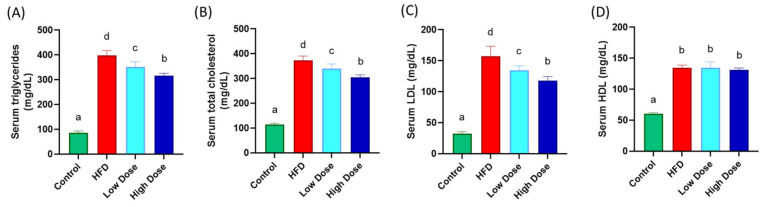
Effects of curcumin–flaxseed oil mixture on the serum lipid profiles of hamsters after the 8-week experimental period. (**A**) Serum TG; (**B**) serum TC; (**C**) serum LDL-C; (**D**) serum HDL-C. Control: normal diet; HFD: high-fat/high-cholesterol diet. Low- and high-dose groups received HCD with supplement (46.84 and 140.53 mg/kg/day, respectively). Values are presented as mean ± SD (*n* = 8 per group). Statistical analysis was performed using one-way ANOVA followed by Tukey’s post hoc test. Different letters indicate significant differences among groups (*p* < 0.05).

After an 8-week HFD intervention, serum TG, TC, LDL-C, and HDL-C levels in the Control, HFD, low-dose, and high-dose groups increased significantly (*p* < 0.05 for all) (Figure 3A–D). Under high-fat diet conditions, supplementation with the curcumin–flaxseed oil mixture significantly reduced serum TG, TC, and LDL-C levels (*p* < 0.05 for all). Compared with the HFD group, the low-dose group showed reductions of 11.9%, 9.0%, and 14.7% in serum TG, TC, and LDL-C levels, respectively, whereas the high-dose group exhibited greater reductions of 20.8%, 18.0%, and 25.0% (*p* < 0.0001). No changes were found in serum HDL-C levels in each group.

### 3.4. Effects of Curcumin–Flaxseed Oil Mixture on Hepatic Lipid

**Figure 4:**
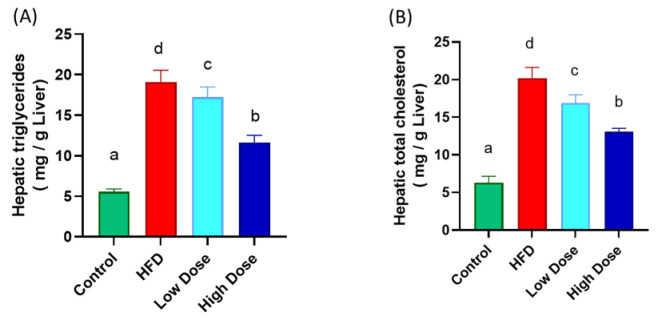
Effects of curcumin–flaxseed oil mixture on hepatic TG (**A**) and TC (**B**) levels in hamsters after 8 weeks of treatment. Control: normal diet; HFD: high-fat/high-cholesterol diet. Low- and high-dose groups received HCD with supplement (46.84 and 140.53 mg/kg/day, respectively). Values are expressed as mean ± SD (*n* = 8 per group). Statistical significance was determined by one-way ANOVA followed by Tukey’s test, and different letters indicate *p* < 0.05.

After an 8-week intervention, hepatic TG and TC levels showed a significant increase in the HFD group than the Control group (3.41- and 3.19-fold, respectively; *p* < 0.0001 for both). Supplementation with curcumin–flaxseed oil mixture significantly decreased hepatic lipid accumulation, with reductions of 9.8% and 16.3% in the low-dose group and 39.2% and 35.3% in the high-dose group for TG and TC, respectively (*p* < 0.0001), indicating a dose-dependent effect (Figure 4A,B).

### 3.5. Effects of a Curcumin–Flaxseed Oil Mixture on Fecal Triglyceride and Total Cholesterol

**Figure 5:**
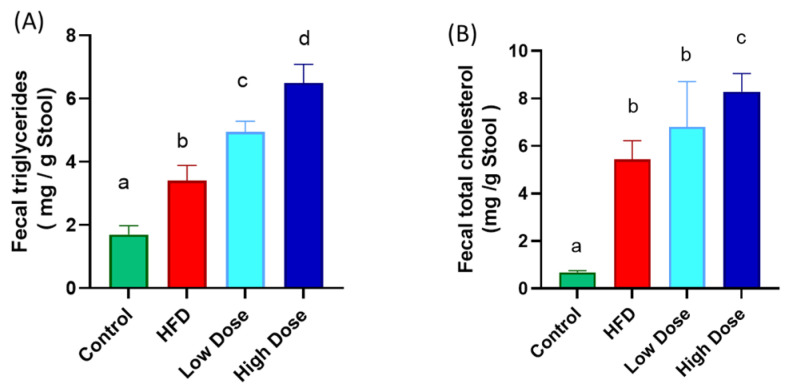
Effects of curcumin–flaxseed oil mixture on fecal TG (**A**) and TC (**B**) excretion in hamsters after 8 weeks of treatment. Control: normal diet; HFD: high-fat/high-cholesterol diet. Low- and high-dose groups received HCD with supplement (46.84 and 140.53 mg/kg/day, respectively). Values are expressed as mean ± SD (*n* = 8 per group). Statistical significance was determined by one-way ANOVA followed by Tukey’s test, and different letters indicate *p* < 0.05.

The results showed that fecal triglyceride and cholesterol excretion were significantly higher in the curcumin–flaxseed oil groups than in both the high-fat diet group and the control group, and the high-dose group exhibited greater excretion than the low-dose group. These findings represented an opposite trend to hepatic triglyceride and cholesterol contents, such that decreased hepatic TG and TC levels were accompanied by increased fecal TG and TC excretion (Figure 5A,B).

### 3.6. Effects of Curcumin–Flaxseed Oil Mixture on Hepatic mRNA Expression

**Figure 6:**
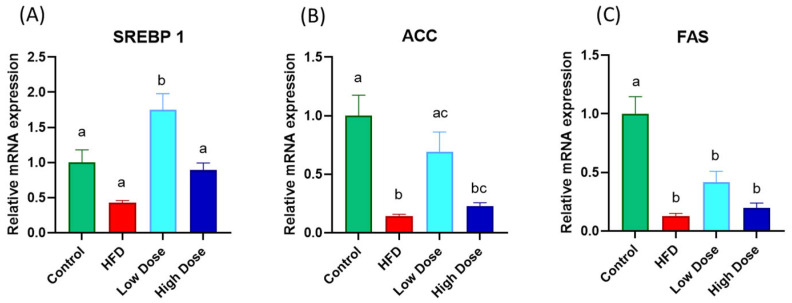
Effects of curcumin–flaxseed oil mixture on hepatic gene expression in hamsters after 8 weeks of treatment. (**A**) SREBP1 (**B**) ACC (**C**) FAS. Control: normal diet; HFD: high-fat/high-cholesterol diet. Low- and High-dose groups received HCD with supplement (46.84 and 140.53 mg/kg/day, respectively). Values are expressed as mean ± SD (*n* = 8 per group). Statistical significance was determined by one-way ANOVA followed by Tukey’s test, and different letters indicate *p* < 0.05.

The hepatic SREBP1 gene expression was significantly higher in the low-dose group than in the Control and HFD groups. Compared with the HFD group, ACC gene expression was significantly increased in the low-dose group. In contrast, for FAS gene expression, no differences were observed among the HFD-fed groups. These findings are limited to transcriptional measurements, and functional lipogenic flux was not assessed. The data are represented in Figure 6.

### 3.7. Effects of a Curcumin–Flaxseed Oil Mixture on Lipidomic Profiles

#### 3.7.1. Serum Diacylglycerol and Triacylglycerol Profile

**Figure 7:**
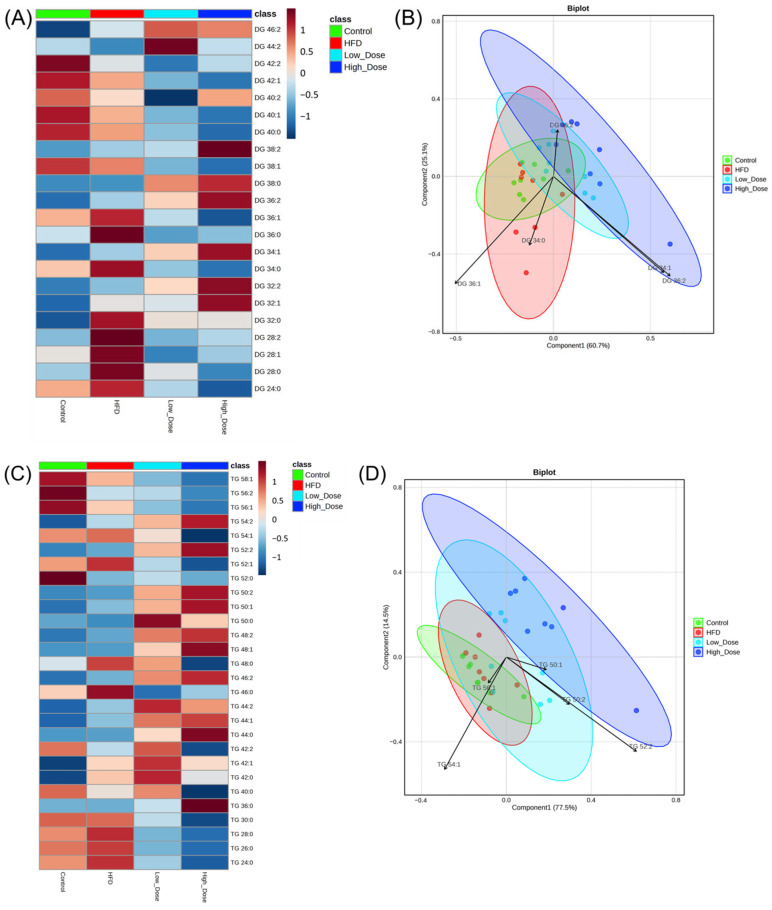
Effects of curcumin–flaxseed oil supplementation on serum diacylglycerol (DG) and triacylglycerol (TG) profiles in hamsters fed a high-fat, high-cholesterol diet. (**A**) Heatmap of differentially distributed serum DG species among the experimental groups. (**B**) Biplot of serum DG species showing group separation and the contribution of discriminating lipid species. (**C**) Heatmap of differentially distributed serum TG species among the experimental groups. (**D**) Biplot of serum TG species showing group separation and the contribution of discriminating lipid species. Lipid species are annotated using the format “lipid class total carbon number:total number of double bonds.” For example, DG 34:1 represents a diacylglycerol species containing a total of 34 carbon atoms and 1 double bond across its fatty acyl chains. These annotations indicate sum-composition lipid species and do not define the exact fatty acyl chain composition unless further structural confirmation is available.

Serum lipidomic analysis revealed distinct group-dependent patterns in diacylglycerol (DG) and triacylglycerol (TG) species. The heatmap showed differential abundance of multiple DG and TG species among the experimental groups. In the biplot, the high-dose group was clearly separated from the HFD group, whereas the low-dose group showed an intermediate distribution. DG 34:1 and DG 36:2 were more closely associated with the high-dose group, while DG 36:1 and DG 34:0 were more aligned with the HFD-related pattern. These findings indicate that curcumin–flaxseed oil supplementation remodeled the circulating DG profile in hamsters fed a high-fat, high-cholesterol diet (Figure 7A,B). In the biplot, the high-dose group was clearly separated from the HFD group, whereas the low-dose group showed an intermediate distribution. TG 54:2, TG 52:2. TG50:2 and TG 50:1 were more closely associated with the high-dose group, while TG 54:1, TG56:1 and TG 58:1 were more aligned with the HFD-related pattern (Figure 7C,D). Such findings indicate that curcumin–flaxseed oil supplementation remodeled the circulating DG and TG profile in hamsters fed a high-fat, high-cholesterol diet.

#### 3.7.2. Hepatic Diacylglycerol and Triacylglycerol Profile

**Figure 8:**
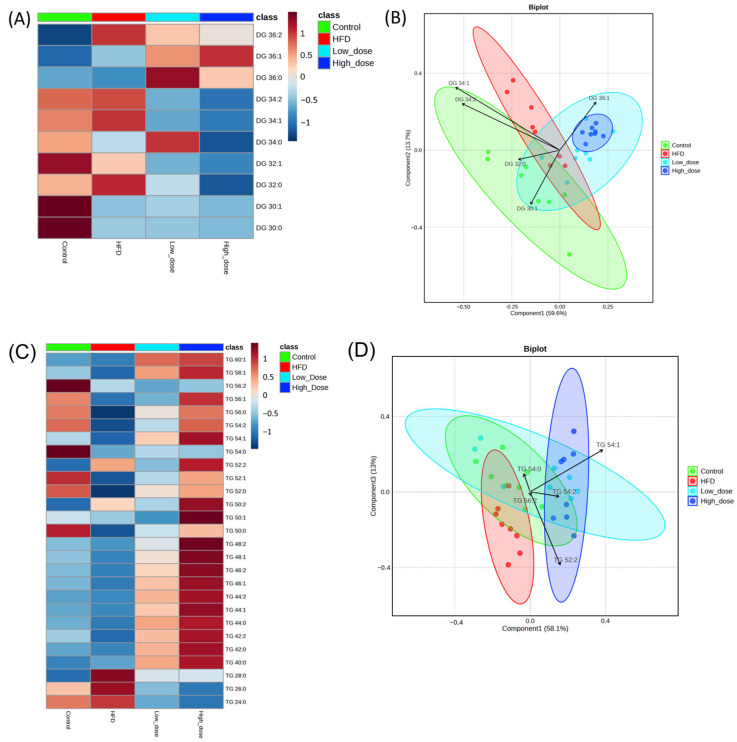
Effects of curcumin–flaxseed oil supplementation on hepatic diacylglycerol (DG) and triacylglycerol (TG) profiles in hamsters fed a high-fat, high-cholesterol diet. (**A**) Heatmap of differentially distributed hepatic DG species among the control, HFD, low-dose, and high-dose groups. (**B**) Biplot of hepatic DG species showing group separation and the contribution of discriminating lipid species. (**C**) Heatmap of differentially distributed hepatic TG species among the control, HFD, low-dose, and high-dose groups. (**D**) Biplot of hepatic TG species showing group separation and the contribution of discriminating lipid species.

The heatmap (Figure 8A) and PLS biplot analysis (Figure 8B) revealed distinct differences in hepatic diacylglycerol (DG) profiles among the Control, HFD, low-dose, and high-dose groups. Lipid species are annotated as “lipid class total carbon number:total number of double bonds.” For example, DG 36:2 represents a DG species with a total of 36 carbon atoms and 2 double bonds across its fatty acyl chains. Compared with the Control group, the HFD group showed higher levels of DG 36:2, DG 34:2, DG 34:1, and DG 32:0, but lower levels of DG 36:0, DG 30:1, and DG 30:0. The low-dose group was characterized by increased DG 36:0, DG 36:1, and DG 34:0, whereas the high-dose group showed higher DG 36:1 and lower levels of several DG species, including DG 34:2, DG 34:1, DG 34:0, DG 32:1, and DG 32:0. These findings suggest that both low- and high-dose supplementation modified hepatic DG profiles, with the high-dose group showing a reduction in several DG species that were elevated under HFD feeding. Consistently, the PLS biplot showed clear group separation, with Component 1 and Component 2 containing 59.6% and 13.7% of the total variance, respectively. The low-dose and high-dose groups were shifted away from the HFD group, indicating that the intervention altered the HFD-induced hepatic DG profile (Figure 8).

The heatmap (Figure 8C) and PLS biplot analysis (Figure 8D) revealed distinct differences in hepatic triacylglycerol (TG) profiles among the Control, HFD, low-dose, and high-dose groups. Compared with the Control group, the HFD group showed higher levels of TG 28:0 and TG 26:0, but lower levels of TG 56:0, TG 52:0, and TG 50:0. After 8 weeks of curcumin–flaxseed oil supplementation, the low-dose group was characterized by increased levels of TG 58:1, TG 56:1, and TG 54:1, whereas the high-dose group showed higher levels of TG 58:1, TG 56:1, TG 54:1, TG 50:1, TG 48:2, TG 48:1, and TG 44:1, together with lower levels of TG 56:2 and TG 54:0. These findings suggest that curcumin–flaxseed oil supplementation modified hepatic TG profiles, with the high-dose group showing broader TG remodeling than the low-dose group.

Lipid species are annotated using the format “lipid class total carbon number:total number of double bonds.” For example, DG 36:2 represents a diacylglycerol species containing a total of 36 carbon atoms and 2 double bonds across its fatty acyl chains. These annotations indicate sum-composition lipid species and do not define the exact fatty acyl chain composition unless further structural confirmation is available.

**Figure 9:**
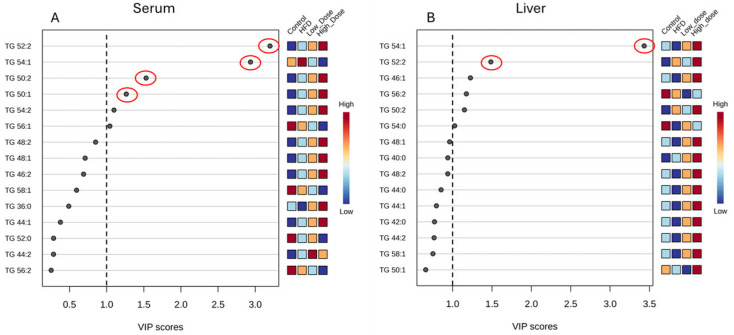
VIP score plots of serum and hepatic TG species. Variable importance in projection (VIP) score plots showing the triacylglycerol (TG) species contributing to group discrimination in serum (**A**) and liver (**B**) among the Control, HFD, low-dose, and high-dose groups. The dashed vertical line indicates a VIP score of 1.0, and TG species with VIP scores greater than 1.0 were considered important contributors to the lipidomic separation. The small heatmaps on the right indicate the relative abundance of each TG species across groups, with red representing higher abundance and blue representing lower abundance. The red circle indicates the area with a higher content of this lipid species.

The VIP score plots further identified the TG species that contributed most strongly to the discrimination among the Control, HFD, low-dose, and high-dose groups. A VIP score greater than 1.0 was considered to indicate an important contribution to group separation. In the serum TG profile (Figure 9A), TG 52:2 and TG 54:1 showed the highest VIP scores, followed by TG 50:2 and TG 50:1, indicating that these TG species were the major contributors to serum TG profile differences among groups. Several additional TG species, including TG 54:2, also showed VIP scores around or above 1.0, suggesting their potential contribution to serum lipidomic variation.

In the liver TG profile (Figure 9B), TG 54:1 showed the highest VIP score, followed by TG 52:2, indicating that these two hepatic TG species were the most influential variables distinguishing the experimental groups. Overall, the VIP score analysis suggests that TG 54:1 and TG 52:2 are key TG species associated with lipidomic differences in both serum and liver. These findings further support that curcumin–flaxseed oil supplementation was associated with remodeling of TG profiles under HFD-induced metabolic disturbance.

## 4. Discussion

Our findings demonstrate that a curcumin–flaxseed oil complex effectively mitigates dyslipidemia in Syrian hamsters subjected to a high-fat, high-cholesterol challenge. This intervention significantly attenuated hepatic steatosis—marked by reduced triglyceride and total cholesterol sequestration—while simultaneously enhancing fecal lipid clearance. Furthermore, the observed shifts in hepatic and serum lipidomic signatures underscore a systemic restoration of lipid homeostasis. Syrian hamsters were selected for this study due to their human-like lipoprotein profiles and low basal rates of endogenous cholesterol synthesis, providing a clinically relevant framework for diet-induced metabolic stress [12].

The reduction in hepatic TG and TC contents, together with the increase in fecal TG and TC excretion, suggests that the curcumin–flaxseed oil mixture may alleviate hepatic lipid burden partly through enhanced lipid elimination. This inverse pattern between hepatic lipid accumulation and fecal lipid output indicates that the intervention may influence whole-body lipid handling rather than simply lowering circulating lipid levels. Curcumin has been reported to improve hepatic steatosis by suppressing pathways involved in de novo lipogenesis and cholesterol synthesis while attenuating hepatic inflammatory signaling [7,20]. Flaxseed oil, which is rich in α-linolenic acid, has also been shown to improve lipid homeostasis and hepatic cholesterol metabolism in high-fat-fed animals [21]. Therefore, the lipid-lowering effect observed in the present study may reflect complementary actions of curcumin and flaxseed oil on hepatic lipid metabolism.

A major strength of this study is the application of lipidomics to complement conventional biochemical measurements. Mass spectrometry-based lipidomics provides a broader view of lipid remodeling than traditional indices such as TG and TC alone [22]. In the present study, hepatic lipidomics revealed marked remodeling of diacylglycerol (DG) species after curcumin–flaxseed oil supplementation. The heatmap showed distinct abundance patterns across DG species, and the biplot demonstrated separation between the intervention groups and the HFD group, particularly in the high-dose group. These findings suggest that the intervention altered not only bulk hepatic lipid content but also DG composition at the molecular level. This observation is biologically meaningful because DGs are central intermediates linking fatty acid esterification, triglyceride synthesis, and lipid signaling, and hepatic DG accumulation has been closely associated with steatosis and metabolic dysfunction [23,24,25]. Thus, the DG remodeling observed here may reflect altered hepatic lipid turnover and storage, which is consistent with the reductions in hepatic TG and TC levels.

Lipidomic profiling revealed distinct alterations in DG and TG species in hamsters fed a high-fat, high-cholesterol diet, supporting the potential involvement of these lipid classes in diet-induced metabolic dysregulation. The PLS-DA analysis showed clear separation between the Control and HFD groups, whereas low-dose and high-dose curcumin–flaxseed oil supplementation shifted lipidomic signatures toward a pattern more distinct from the HFD group. These findings suggest that the curcumin–flaxseed oil mixture modulated lipidomic profiles associated with hepatic lipid accumulation and systemic lipid handling. In the liver, high-dose supplementation reduced several DG species, including DG 34:2, DG 34:1, DG 32:1, and DG 32:0, suggesting partial attenuation of HFD-associated hepatic DG accumulation or remodeling. Serum lipidomic analysis further showed that curcumin–flaxseed oil supplementation remodeled circulating DG species in hamsters fed a high-fat, high-cholesterol diet. The serum heatmap demonstrated differential abundance patterns of multiple DG species among groups, and the biplot showed that the high-dose group was more clearly separated from the HFD group, whereas the low-dose group exhibited an intermediate distribution. In particular, serum DG 34:1 and DG 36:2 were more closely associated with the high-dose group, whereas DG 36:1 and DG 34:0 were more aligned with the HFD-related pattern. Notably, changes in DG molecular species observed in lipidomic may reflect alterations in sum-composition lipid profiles rather than definitive changes in specific fatty acyl chain composition. Therefore, the higher abundance of serum DG 34:1 and DG 36:2 in the high-dose group is more appropriately interpreted as selective remodeling of circulating DG composition rather than direct evidence of either beneficial or adverse metabolic effects, because the biological significance of individual DG species is highly species-specific and context-dependent [26]. Since DGs are key intermediates linking fatty acid esterification, triglyceride synthesis, and lipid signaling, the remodeling of both hepatic and circulating DG species may reflect changes in lipid turnover, storage, and systemic lipid handling [26,27]. Taken together with the reductions in hepatic TG and TC contents observed in the intervention groups, these findings support the view that the curcumin–flaxseed oil mixture modulated lipid homeostasis at both hepatic and systemic levels. However, complete normalization of hepatic lipid metabolic flux cannot be inferred from the current lipidomic data alone.

Although we originally expected the intervention to suppress lipogenic signaling, hepatic SREBP-1 and ACC mRNA expression were increased in the low-dose group, whereas FAS expression was not significantly altered. This pattern suggests that regulation of the de novo lipogenesis pathway was not uniformly changed and that individual lipogenic genes may respond differently to dietary intervention. SREBP-1 is a key upstream transcription factor involved in hepatic lipogenesis, ACC is an early rate-limiting enzyme in fatty acid synthesis, and FAS is a downstream enzyme responsible for long-chain fatty acid synthesis [28]. Therefore, the increase in SREBP-1 and ACC in the absence of a significant change in FAS may reflect differential regulation within the lipogenic pathway rather than generalized activation of hepatic lipid synthesis. Interestingly, SREBP-1 expression in the high-dose group was lower than that in the low-dose group. This finding may be associated with the higher intake of *n*-3 fatty acids, particularly ALA, in the high-dose treatment. Previous studies have demonstrated that *n*-3 polyunsaturated fatty acids suppress hepatic SREBP-1 activity and inhibit de novo lipogenesis, thereby reducing hepatic lipid accumulation [29]. Therefore, the lower SREBP-1 expression observed in the high-dose group may reflect a stronger inhibitory effect of *n*-3 fatty acids on hepatic lipogenic signaling. In addition, the biological activity of SREBP-1 is regulated not only at the transcriptional level but also through precursor cleavage, maturation, and nuclear translocation; thus, increased SREBP-1 mRNA expression does not necessarily indicate enhanced lipogenic flux [30]. Similarly, ACC activity can be regulated post-translationally, suggesting that mRNA expression alone may not directly reflect functional lipid synthesis. Curcumin and flaxseed oil have been reported to reduce hepatic lipid accumulation, enhance fatty acid β-oxidation, suppress hepatic lipogenic enzyme activities, and increase fecal neutral sterol excretion in high-fat-fed hamsters or rats [14,15,31], supporting the possibility that the low-dose response reflects partial metabolic adaptation and improved lipid handling rather than true activation of hepatic lipid deposition. Given that hepatic TG and TC contents were reduced despite the elevation of SREBP-1 and ACC, these changes may represent a compensatory or adaptive response to altered hepatic lipid handling rather than a direct signal promoting lipid accumulation. Overall, these findings suggest that the regulation of de novo lipogenesis may be influenced by multiple factors, including dietary composition, cholesterol load, feeding duration, and metabolic status.

**Figure 10:**
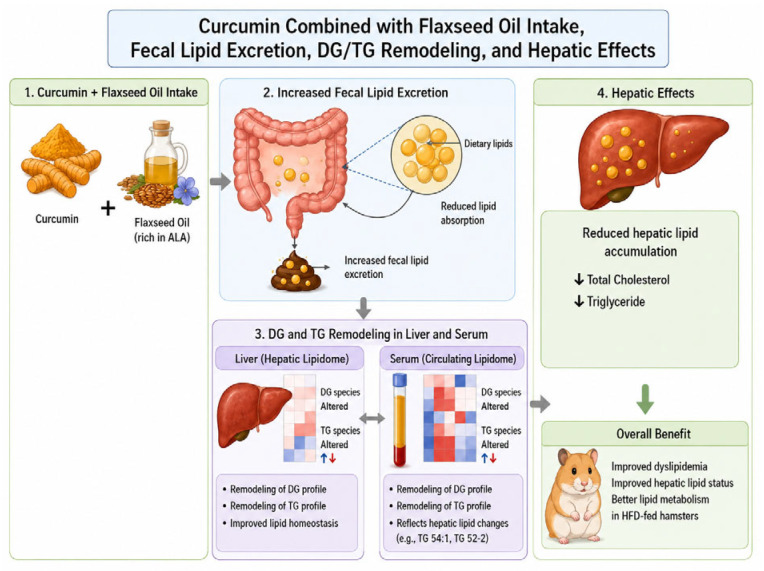
Schematic summary of the effects of curcumin combined with flaxseed oil on fecal lipid excretion, DG/TG remodeling, and hepatic lipid accumulation in HFD-fed hamsters. Curcumin combined with flaxseed oil, which is rich in alpha-linolenic acid (ALA), increased fecal lipid excretion and may reduce dietary lipid absorption. These effects were accompanied by remodeling of hepatic and serum diacylglycerol (DG) and triacylglycerol (TG) profiles. Overall, curcumin combined with flaxseed oil was associated with reduced hepatic lipid accumulation, including lower hepatic total cholesterol and triglyceride levels, and improved dyslipidemia and hepatic lipid status in HFD-fed hamsters. Arrows indicate the proposed direction of metabolic changes.

Our findings revealed that curcumin–flaxseed oil supplementation reversed obesity-associated MASLD and related metabolic derangements. Curcumin–flaxseed oil supplementation leads to rapid metabolic improvements through alterations in lipid profiles and selected hormonal adaptations. Curcumin–flaxseed oil supplementation sustains and amplifies these improvements by promoting metabolic flexibility and reducing hepatic lipid overload, and attenuating inflammation [15,32]. Lipidomic profiling supported the aforementioned findings. Low-dose curcumin–flaxseed oil supplementation partially remodeled lipid metabolism, whereas high-dose supplementation induced lipidomic changes that differed from those observed in the low-dose group. In the circulating DG profile, DG 34:1 and DG 36:2 was more closely associated with the high-dose group, suggesting remodeling of circulating DG species after curcumin–flaxseed oil supplementation. High-dose supplementation was also associated with shifts in serum and hepatic lipid profiles, accompanied by reduced lipid accumulation. These results suggest that curcumin–flaxseed oil supplementation modulates hepatic and circulating lipid metabolism in a dose-related manner (Figure 10).

Furthermore, nutritional evidence suggests that a reduced linoleic acid to α-linolenic acid ratio mitigates weight gain and metabolic dysfunction in obese cohorts [33]. The therapeutic potential of *n*-3 PUFAs is particularly salient, as they optimize lipid metabolism and systemic inflammatory status [34]. Mechanistically, *n*-3 PUFAs antagonize pro-inflammatory pathways, such as NF-κB, while modulating PPARγ signaling to enhance insulin sensitivity and resolve chronic low-grade inflammation [35]. Collectively, optimizing the *n*-6/*n*-3 fatty acid balance represents a pivotal nutritional strategy for the management of metabolic disease.

In the present study, hepatic FFA signatures—characterized by mass spectrometry—revealed a profound metabolic adaptation after a specific dietary intervention. The observed normalization of TG profiles not only reflects the re-establishment of lipid homeostasis but also identifies specific lipid species as key biomarkers of the adaptive metabolic response following dietary intervention. These findings suggest that monitoring circulating TG levels could enable more precise assessments of postoperative health and therapeutic success, providing a minimally invasive window into the resolution of MASLD [36].

Several limitations should be acknowledged. First, this study lacked curcumin-only and flaxseed oil-only groups, which prevents us from distinguishing the individual contribution and optimal dose of each component; therefore, the findings should be interpreted as the effects of the combined curcumin and flaxseed oil formulation. Future studies with separate and combined intervention groups are needed to clarify their individual and combined effects. Second, the apparent lipogenesis paradox may reflect the limitation of using lipogenic mRNA expression as a direct indicator of lipid synthesis. Although SREBP-1 and ACC mRNA levels were increased after intervention, hepatic TG and TC contents were reduced, suggesting adaptive regulation of hepatic lipid metabolism rather than enhanced lipid accumulation. Because SREBP-1 and ACC are also regulated post-transcriptionally and post-translationally, mRNA expression alone cannot confirm increased lipogenic flux. The reduced hepatic lipid content, together with increased fecal lipid excretion, may partially support reduced net hepatic lipid retention after intervention. Third, the interpretation of cholesteryl ester (CE) species is limited in the present lipidomic analysis because CE species showed a high proportion of missing values, possibly due to low abundance or limited detection sensitivity under the current analytical conditions; therefore, we focused mainly on TAG and DAG species, which were more consistently detected and showed clearer intervention-related changes. Although lipidomic profiling provided a broader view of lipid remodeling, it did not establish direct causal relationships between individual DG species and the observed metabolic improvements. Finally, the underlying mechanisms were inferred mainly from biochemical and lipidomic findings and were not fully validated at the protein or enzyme-activity level. Nevertheless, the present results suggest that combined supplementation with curcumin and flaxseed oil may be associated with favorable modulation of hepatic and circulating lipid metabolism under high-fat, high-cholesterol dietary conditions.

## 5. Conclusions

In conclusion, curcumin combined with flaxseed oil improved dyslipidemia and reduced hepatic lipid accumulation in hamsters fed a high-fat, high-cholesterol diet. These effects were accompanied by increased fecal lipid excretion and remodeling of hepatic and serum DG profiles, suggesting potential lipid remodeling at both the biochemical and molecular levels. A similar trend was observed in serum TG profiles, particularly TG 54:1 and TG 52:2, suggesting that changes in circulating lipids may mirror the hepatic lipidomic response. Overall, these findings suggest that curcumin combined with flaxseed oil may serve as complementary dietary bioactives for attenuating diet-induced disturbances in lipid metabolism, although further studies are needed to confirm the functional significance of these lipidomic changes.

## Figures and Tables

**Table 1:** Composition of the experimental diets.

Ingredient (g/kg Diet)	Normal Diet	High Fat Diet
Casein	140	140
Corn starch	620.7	558.7
Sucrose	100	100
Cellulose	50	50
Soybean oil	40	40
Lard	0	110
Mineral mixture	35	35
Vitamin mixture	10	10
L-cysteine	1.8	1.8
Choline bitartrate	2.5	2.5
Cholesterol	0	2.1
Total weight (g)	1000	1050
Total calorie (kcal)	3903	4645

**Table 2:** Primer sequences used for quantitative real-time PCR analysis.

Gene Name	Primer Sequence (5′ → 3′)
β-actin-F	GTGGATCAGCAAGCAGGAGT
β-actin-R	GGGTGTAAAACGCAGCTCAG
Srebp1-F	CTTCTTACAGCACAGCAACCA
Sebp1-R	TTCACACCCTCCATAGTCACA
Acc1-F	TCGTGAAGGGCTACCTCTAA
Acc1-R	CCACAATGTAAGCGCCAAAC
FAS-F	ACACGCTAGAGGGGGAGAAT
FAS-R	GCCGTGGTGTGTAGTCTTGA

## Data Availability

The original contributions presented in this study are included in the article/Supplementary Material. Further inquiries can be directed to the corresponding authors.

## Supplementary Materials

The following supporting information can be downloaded at: https://www.mdpi.com/article/10.3390/nu18111747/s1, Figure S1: Validation of PLS-DA models based on serum DG profiles; Figure S2: Validation of PLS-DA models based on hepatic DG profiles; Figure S3: Validation of PLS-DA models based on serum TG profiles; Figure S4: Validation of PLS-DA models based on hepatic TG profiles; Table S1: Variable importance in projection (VIP) scores and fold-change analysis of lipid species identified from PLS-DA models.

## Abbreviations

The following abbreviations are used in this manuscript:

ACC	acetyl-CoA carboxylase
ALA	α-linolenic acid
CVD	Cardiovascular disease
DG	Diacylglycerol
DNL	de novo lipogenesis
FAS	fatty acid synthase
HDL	High-density lipoprotein cholesterol
HFD	High-fat diet
LDL	Low-density lipoprotein cholesterol
MASLD	metabolic dysfunction-associated steatotic liver disease
PC	Phosphatidylcholine
PLS	Partial least squares
PS	Phosphatidylserine
SREBP-1	Sterol Regulatory Element Binding Protein
TG	Triglyceride
