# Supplementary material for: Curcumin Combined with Flaxseed Oil Modulates Lipid Metabolism in Hamsters Fed a High-Fat, High-Cholesterol Diet: Insights from Lipidomics
Source: Nutrients. 2026 May 29;18(11):1747. doi: 10.3390/nu18111747 (PMC13259485; doi:10.3390/nu18111747)
Supplement: Supplementary file 1 [file nutrients-18-01747-s001.zip › nutrients-4290301-supplementary.pdf]

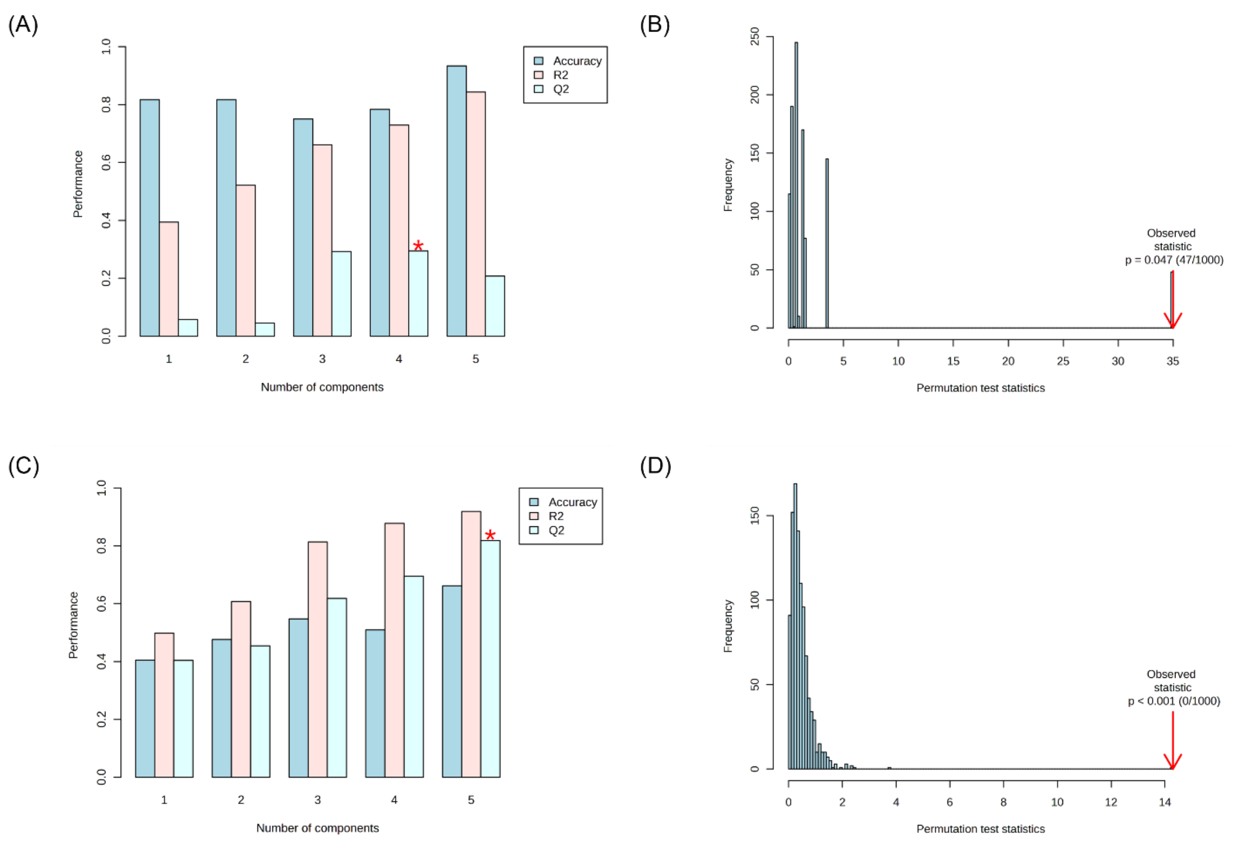

Supplementary Figure S1. Validation of PLS-DA models based on serum DG profiles.
Cross-validation and permutation testing were performed for PLS-DA models based on serum diacylglycerol (DG) profiles. (A, B) Validation of the model comparing the Control and HFD groups. The optimal model was selected using four components, with $Q^2 = 0.29$ and a permutation test p-value of 0.047. Because the Q^2 value was < 0.4, this model should be interpreted as exploratory despite the significant permutation test result. (C, D) Validation of the model including the Control, HFD, Low-dose, and High-dose groups. The optimal model was selected using five components, with $Q^2 = 0.82$ and a permutation test p-value < 0.001. The red asterisk indicates the optimal component number determined by the highest Q^2 value. The red arrow indicates the observed separation distance in the permutation test.

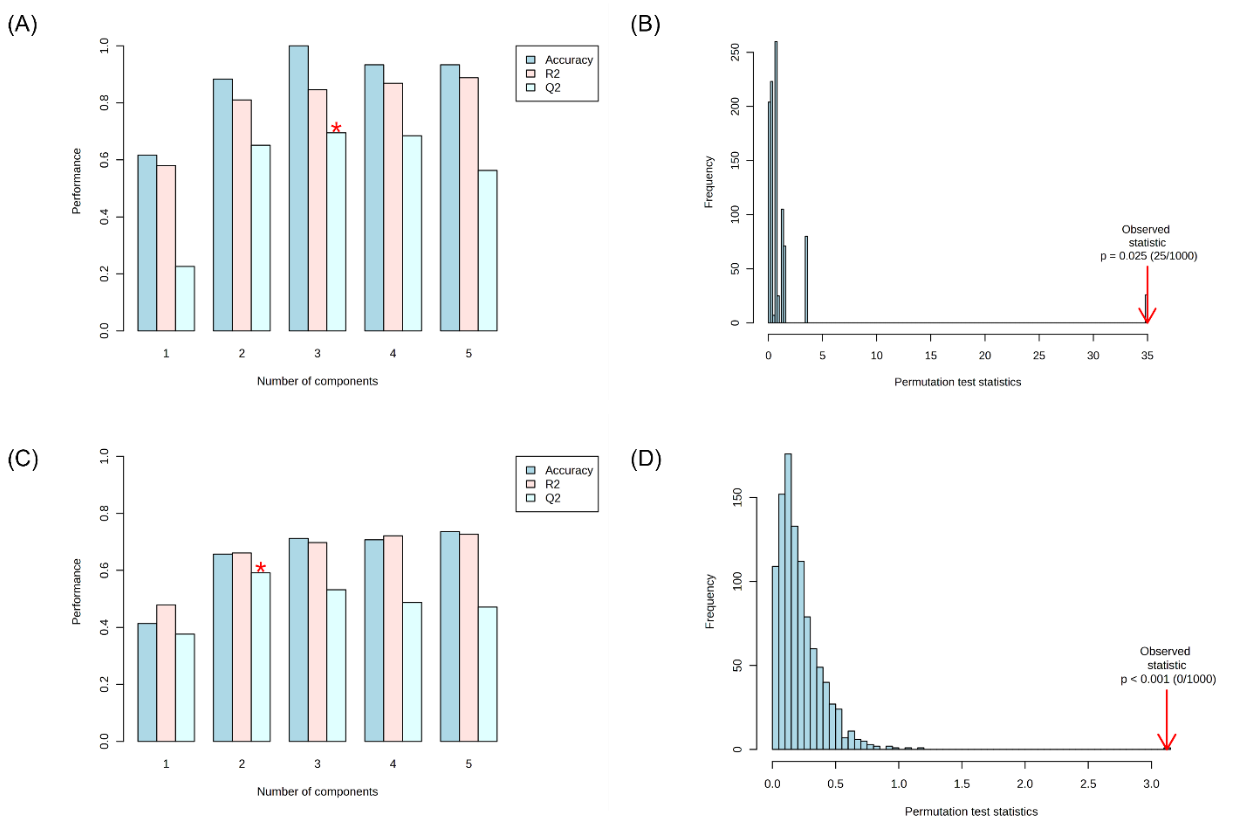

Supplementary Figure S2. Validation of PLS-DA models based on hepatic DG profiles.
Cross-validation and permutation testing were performed for PLS-DA models based on hepatic diacylglycerol (DG) profiles. (A, B) Validation of the model comparing the Control and HFD groups. The optimal model was selected using three components, with $Q^2 = 0.69$ and a permutation test p-value of 0.025. (C, D) Validation of the model including the Control, HFD, Low-dose, and High-dose groups. The optimal model was selected using two components, with $Q^2 = 0.59$ and a permutation test p-value < 0.001. The red asterisk indicates the optimal component number determined by the highest Q^2 value. The red arrow indicates the observed separation distance in the permutation test. Both models met the validation criteria, with $Q^2 > 0.4$ and permutation test p-values < 0.05.

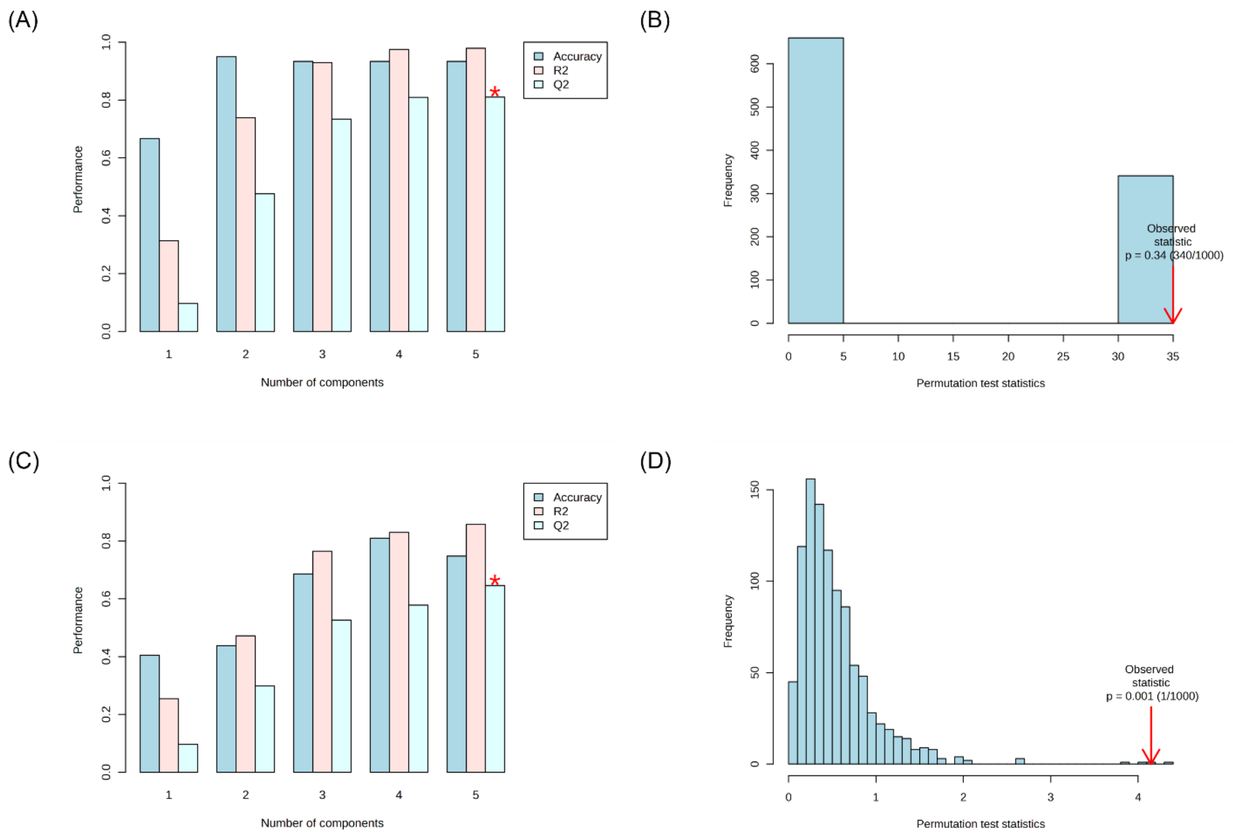

Supplementary Figure S3. Validation of PLS-DA models based on serum TG profiles.
Cross-validation and permutation testing were performed for PLS-DA models based on serum triacylglycerol (TG) profiles. (A, B) Validation of the model comparing the Control and HFD groups. The optimal model was selected using five components, with $Q^2 = 0.81$ and a permutation test p-value of 0.34. Because the permutation test p-value was > 0.05, this model should be interpreted as exploratory despite the high Q^2 value. (C, D) Validation of the model including the Control, HFD, Low-dose, and High-dose groups. The optimal model was selected using five components, with $Q^2 = 0.65$ and a permutation test p-value of 0.001. The red asterisk indicates the optimal component number determined by the highest Q^2 value. The red arrow indicates the observed separation distance in the permutation test.

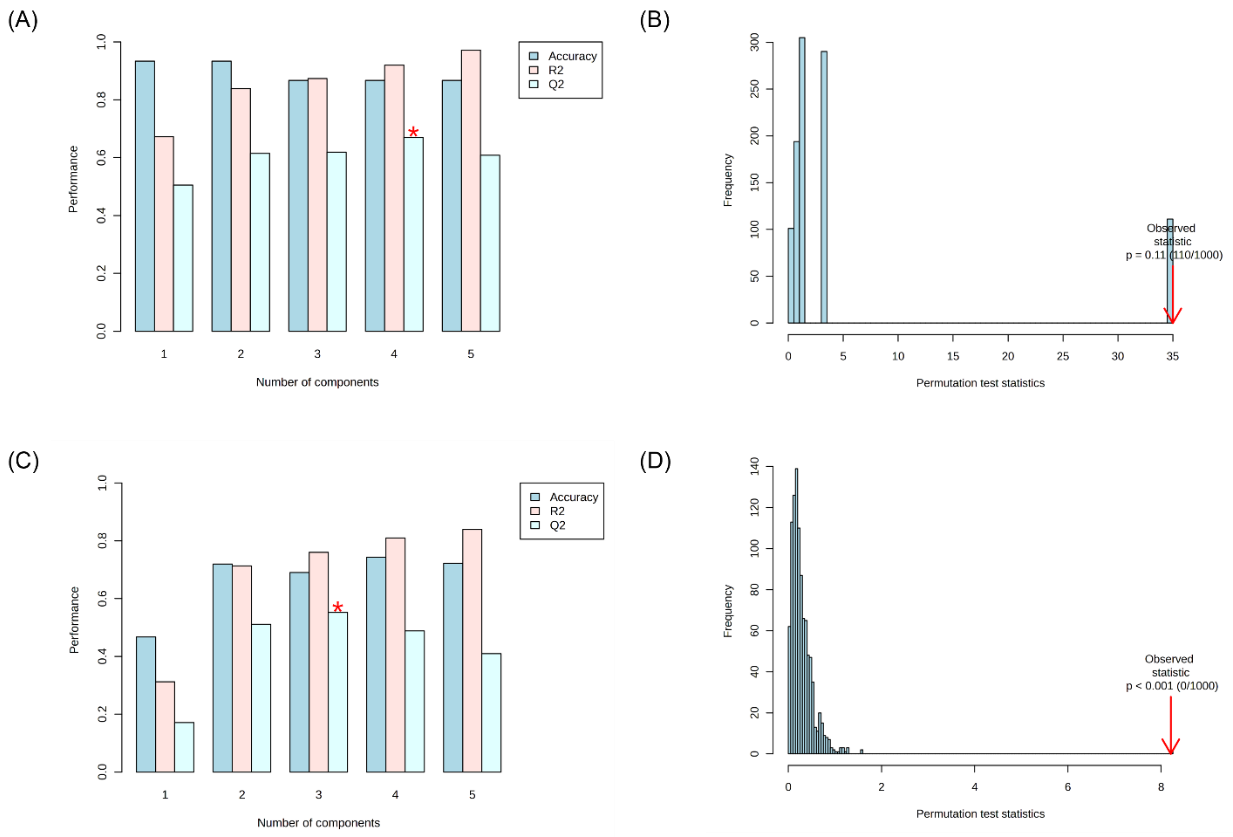

Supplementary Figure S4. Validation of PLS-DA models based on hepatic TG profiles.
Cross-validation and permutation testing were performed for PLS-DA models based on hepatic triacylglycerol (TG) profiles. (A, B) Validation of the model comparing the Control and HFD groups. The optimal model was selected using four components, with $Q^2 = 0.67$ and a permutation test p-value of 0.11. Because the permutation test p-value was > 0.05, this model should be interpreted as exploratory despite the high Q^2 value. (C, D) Validation of the model including the Control, HFD, Low-dose, and High-dose groups. The optimal model was selected using three components, with $Q^2 = 0.55$ and a permutation test p-value < 0.001. The red asterisk indicates the optimal component number determined by the highest Q^2 value. The red arrow indicates the observed separation distance in the permutation test.

Supplementary Table S1. Variable importance in projection (VIP) scores and fold-change analysis of lipid species identified from PLS-DA models.

Sample Type	Lipid species	VIP score	Fold change	p-value	Trend
Liver	DG 30:0	1.05360	0.08861	0.05361	Decrease
Liver	DG 30:1	1.98820	0.06497	0.00230	Decrease
Liver	DG 32:0	0.89234	1.31784	0.05695	Increase
Liver	DG 32:1	0.29007	0.73292	0.31501	Decrease
Liver	DG 34:0	0.54652	0.88660	0.53176	Decrease
Liver	DG 34:1	0.72201	1.13565	0.58068	Increase
Liver	DG 34:2	0.23921	1.04843	0.84556	Increase
Liver	DG 36:0	0.26098	0.92678	0.54933	Decrease
Liver	DG 36:1	0.90420	1.61402	0.01937	Increase
Liver	DG 36:2	1.51450	2.19742	0.00065	Increase
Liver	TG 24:0	0.11224	1.07515	0.77888	Increase
Liver	TG 26:0	0.39042	1.50105	0.10015	Increase
Liver	TG 28:0	0.44721	1.38005	0.26860	Increase
Liver	TG 40:0	0.18283	1.28228	0.30267	Increase
Liver	TG 42:0	0.14388	0.84552	0.44981	Decrease
Liver	TG 42:2	0.23984	0.44269	0.29858	Decrease
Liver	TG 44:0	0.23474	0.86118	0.35029	Decrease
Liver	TG 44:1	0.12103	0.91920	0.68150	Decrease
Liver	TG 44:2	0.11263	0.86460	0.54013	Decrease
Liver	TG 46:1	0.37213	0.90843	0.39337	Decrease
Liver	TG 46:2	0.16001	0.89550	0.60392	Decrease
Liver	TG 48:1	0.31064	0.92601	0.51285	Decrease
Liver	TG 48:2	0.19334	0.93914	0.59147	Decrease
Liver	TG 50:0	0.47698	0.81340	0.33326	Decrease
Liver	TG 50:1	0.17806	0.96984	0.71198	Decrease
Liver	TG 50:2	1.05970	1.29838	0.06170	Increase
Liver	TG 52:0	0.81743	0.63629	0.01056	Decrease
Liver	TG 52:1	1.85510	0.71018	0.00033	Decrease
Liver	TG 52:2	1.95760	1.43002	0.03356	Increase
Liver	TG 54:0	1.57360	0.52211	0.00015	Decrease
Liver	TG 54:1	2.77230	0.82075	0.02158	Decrease
Liver	TG 54:2	1.44580	0.80013	0.09637	Decrease
Liver	TG 56:0	0.84714	0.49254	0.00046	Decrease
Liver	TG 56:1	1.31690	0.75683	0.00368	Decrease
Liver	TG 56:2	1.35960	0.56453	0.00241	Decrease
Liver	TG 58:1	0.51312	0.66966	0.01878	Decrease
Liver	TG 60:1	0.05476	0.93861	0.66895	Decrease
Serum	DG 24:0	0.39036	1.16794	0.02221	Increase
Serum	DG 28:0	0.29501	1.11169	0.04643	Increase
Serum	DG 28:1	0.28403	1.14127	0.08675	Increase
Serum	DG 28:2	0.32843	2.04977	0.14584	Increase
Serum	DG 32:0	1.20640	1.27129	0.00486	Increase
Serum	DG 32:1	0.65311	1.13576	0.12561	Increase
Serum	DG 32:2	0.23983	1.04644	0.14817	Increase
Serum	DG 34:0	1.08520	1.23183	0.31209	Increase
Serum	DG 34:1	1.80310	1.34009	0.18418	Increase
Serum	DG 36:0	1.50720	1.24216	0.02888	Increase

Serum	DG 36:1	2.69950	1.14033	0.07609	Increase
Serum	DG 36:2	2.09340	1.43086	0.13017	Increase
Serum	DG 38:0	0.00331	1.00885	0.98584	Increase
Serum	DG 38:1	0.24284	0.91391	0.17921	Decrease
Serum	DG 38:2	0.10424	1.14668	0.48806	Increase
Serum	DG 40:0	0.32157	0.79233	0.01813	Decrease
Serum	DG 40:1	0.39164	0.74493	0.00149	Decrease
Serum	DG 40:2	0.11704	0.80860	0.38849	Decrease
Serum	DG 42:1	0.43323	0.72982	0.00764	Decrease
Serum	DG 42:2	0.31473	0.90950	0.41104	Decrease
Serum	DG 44:2	0.18614	0.94859	0.67581	Decrease
Serum	DG 46:2	0.77507	1.18174	0.30829	Increase
Serum	TG 24:0	0.22173	1.01824	0.50387	Increase
Serum	TG 26:0	0.10891	1.01290	0.79919	Increase
Serum	TG 28:0	0.22564	1.02243	0.49501	Increase
Serum	TG 30:0	0.01470	0.99731	0.92204	Decrease
Serum	TG 36:0	0.18951	0.49336	0.01782	Decrease
Serum	TG 40:0	0.14558	0.97886	0.43511	Decrease
Serum	TG 42:0	0.13558	1.01084	0.51481	Increase
Serum	TG 42:1	0.34466	1.02630	0.11120	Increase
Serum	TG 42:2	0.19899	0.96469	0.33553	Decrease
Serum	TG 44:0	0.01505	1.00111	0.95984	Increase
Serum	TG 44:1	0.68363	1.04186	0.00102	Increase
Serum	TG 44:2	0.57641	1.06258	0.01676	Increase
Serum	TG 46:0	0.21062	1.01454	0.49706	Increase
Serum	TG 46:2	0.83561	1.07113	0.02826	Increase
Serum	TG 48:0	0.58363	1.04523	0.14047	Increase
Serum	TG 48:1	0.07932	1.00419	0.89141	Increase
Serum	TG 48:2	0.66097	1.04932	0.38547	Increase
Serum	TG 50:0	0.09135	0.98687	0.90225	Decrease
Serum	TG 50:1	1.48010	1.09890	0.19585	Increase
Serum	TG 50:2	0.43028	1.03933	0.83187	Increase
Serum	TG 52:0	1.21060	0.81248	0.08079	Decrease
Serum	TG 52:1	0.18427	1.01391	0.49811	Increase
Serum	TG 52:2	1.98600	1.15564	0.59468	Increase
Serum	TG 54:1	1.60210	1.01839	0.34641	Increase
Serum	TG 54:2	2.68200	1.10122	0.00612	Increase
Serum	TG 56:1	2.38120	0.87161	0.00001	Decrease
Serum	TG 56:2	0.80425	0.92353	0.00262	Decrease
Serum	TG 58:1	1.19280	0.88355	0.00002	Decrease

DG, diacylglycerol; TG, triacylglycerol.
